# FAT4 regulates the EMT and autophagy in colorectal cancer cells in part via the PI3K-AKT signaling axis

**DOI:** 10.1186/s13046-019-1043-0

**Published:** 2019-03-04

**Authors:** Ran Wei, Yuhong Xiao, Yi Song, Huiping Yuan, Jun Luo, Wei Xu

**Affiliations:** 1 Department of Rehabilitation Medicine, The Second Affiliated Hospital of Nanchang University, Nanchang, 330006 Jiangxi China; 2 Department of General Surgery, The Second Affiliated Hospital of Nanchang University, Nanchang, 330006 Jiangxi China; 3 The First Clinical Medical College, Nanchang University, Nanchang, 330006 Jiangxi China

**Keywords:** Colorectal cancer, FAT4, PI3K/AKT, EMT, Proliferation, Autophagy

## Abstract

**Background:**

FAT4 functions as a tumor suppressor, and previous findings have demonstrated that FAT4 can inhibit the epithelial-to-mesenchymal transition (EMT) and the proliferation of gastric cancer cells. However, few studies have investigated the role of FAT4 in the development of colorectal cancer (CRC). The current study aimed to detect the role of FAT4 in the invasion, migration, proliferation and autophagy of CRC and elucidate the probable molecular mechanisms through which FAT4 interacts with these processes.

**Methods:**

Transwell invasion assays, MTT assays, transmission electron microscopy, immunohistochemistry and western blotting were performed to evaluate the migration, invasion, proliferation and autophagy abilities of CRC cells, and the levels of active molecules involved in PI3K/AKT signaling were examined through a western blotting analysis. In addition, the function of FAT4 in vivo was assessed using a tumor xenograft model.

**Results:**

FAT4 expression in CRC tissues was weaker than that in nonmalignant tissues and could inhibit cell invasion, migration, and proliferation by promoting autophagy in vitro. Furthermore, the regulatory effects of FAT4 on autophagy and the EMT were partially attributed to the PI3K-AKT signaling pathway. The results in vivo also showed that FAT4 modulated CRC tumorigenesis.

**Conclusion:**

FAT4 can regulate the activity of PI3K to promote autophagy and inhibit the EMT in part through the PI3K/AKT/mTOR and PI3K/AKT/GSK-3β signaling pathways.

## Introduction

Colorectal cancer (CRC) is a common human malignancy and a leading cause of cancer-related mortality worldwide, and the available statistics suggest that over 1.2 million people are affected by CRC each year [1]. Further investigations of the molecular mechanisms underlying the invasive behaviors of CRC are urgently needed. FAT tumor suppressor homolog 4 (FAT4), which belongs to the FAT protein family (FAT1–4) identified in mammals, was first identified as a cancer suppressor in a mouse mammary epithelial cell line [2, 3]. Although few studies have examined the function of FAT4 in cancer, the relationship between FAT4 expression and tumorigenesis has been revealed in breast and gastric cancers, and a study conducted by Cai demonstrated that FAT4 plays a significant role in inhibiting the epithelial-to-mesenchymal transition (EMT) and the proliferation of human gastric cancer cells [4]. The EMT is essential for numerous developmental processes and is considered a hallmark of the aggressive invasion and metastasis of late-stage tumors [5, 6]. The EMT requires cancer cells to survive independently from the primary tumor site without a nutrient support system, and thus, these cells are more prone to undergo autophagy to gain energy [7].

Autophagy, which is a type of homeostatic mechanism as well as a response to stress, involves the engulfment, digestion and recycling of cellular proteins, organelles and cytoplasmic components through the lysosomal degradation pathway to sustain cellular metabolism. Autophagy might be induced in cancer cells subjected to various stressors, including radiation, hypoxia, and growth factor deprivation [7]. An increase in cell proliferation and a decrease in aerobic glycolysis result in a decreased supply of ATP, and under these conditions, autophagy plays a significant role in energy production [8]. However, the effect of autophagy on cancer cells remains controversial [9]. Autophagy might protect the genome from damage and inhibit tumorigenesis due to its function as a cancer suppressor, but this process might also activate metabolic stress responses to promote tumor growth [10, 11]. Some studies have suggested that autophagy can help cells survive, but other evidence shows that unrestrained autophagy might cause cell death [12, 13]. However, the exact contribution of autophagy to the EMT in CRC remains unclear. The studies conducted by Li and Zhu have shown that the energy provided by autophagy can promote the invasion of cancer cells by inducing the EMT in hepatocellular carcinoma and pancreatic cancer [14, 15]. However, Gugnoni’s research group revealed that autophagy might negatively control the EMT in papillary thyroid carcinoma [16]. In addition, the inhibition of autophagy might exert some effects on the EMT, and the molecular and cellular functions of FAT4 in autophagy require further analysis. The mechanism underlying the progression of autophagy is complicated, and the phosphoinositide 3-kinase (PI3K)/serine/threonine protein kinase B (AKT) pathway plays a significant role in the inhibition of autophagic progression.

The abnormal activation of PI3K, which belongs to a conserved family of lipid kinases that phosphorylate the 30-hydroxyl group of phosphoinositides [17], is very frequently observed in human cancer. This protein phosphorylates PIP2 to produce PIP3 by regulating the extracellular levels of protein kinases, such as ERK, and epidermal growth factor receptor (EGFR) [18, 19]. PIP3 is an important second messenger involved in the recruitment of AKT, which signals mTOR or GSK-3β and is involved in the activation of growth, proliferation and survival signaling responses.

In the present study, we examined the efficacy of the FAT4-induced promotion of the formation of autophagosomes to increase autophagy in vitro. We further assessed the tumor autophagy-promoting mechanism of FAT4 by decreasing the activity of the PI3K/AKT/mTOR signaling pathway in CRC and by inhibiting the EMT through reductions in the levels of proteins involved in the PI3K/AKT/GSK-3β signaling pathway. The results of these experiments should provide novel insights into the effects of FAT4 and PI3K/AKT signaling on tumor therapy.

## Materials and methods

### Tissue specimens

In our study, 100 formalin-fixed, paraffin-embedded CRC samples and paired adjacent benign tissues were randomly collected from 72 men and 28 women with a mean age of 60 years at the Second Affiliated Hospital of Nanchang University, Nanchang, China. Informed consent was obtained from the study participants prior to their participation, and the study was highly supported by the Medical Research Ethics Committee of the Second Affiliated Hospital of Nanchang University.

### Cell culture

Human CRC cell lines (HCT116, LOVO and SW480) from the Chinese Type Culture Collection were cultured in RPMI 1640 medium (RPMI 1640, Gibco, USA) supplemented with 10% fetal bovine serum (FBS, Gibco, USA) and 2% penicillin/streptomycin (HyClone, Shanghai, China). All the cell lines were incubated under standard conditions at 37 °C in an atmosphere contaning 5% CO2.Once the cells reached approximately 80% confluence, they were passaged by trypsinization.

For the induction and inhibition of autophagy, the cells were treated with 250 nM Torin 1 (Sigma Aldrich, Missouri, USA) and 2 μM MHY1485 (Sigma Aldrich, Missouri, USA), respectively, and to regulate PI3K activity, the cells were treated with 150 nM wortmannin (Sigma Aldrich, Missouri, USA) and 50 μg/mL 740Y-P (Cayman, Michigan, USA).

### Quantitative RT-PCR

We utilized the TRIzol reagent (Invitrogen, Carlsbad, CA, USA) for the extraction of total RNA from tissue samples and cultured cells. Quantitative RT-PCR was performed using a PrimeScript RT reagent kit (TaKaRa, Dalian, China) and SYBR Premix Ex Taq (TaKaRa, Dalian, China). The following primer sequences were selected: FAT4, forward, 5’-TTGAAGGAAGGAGAACCCAT-3′, and reverse, 5’-TCGTCCAATAGTAAAGAGGC-3′.

### Immunohistochemistry

The CRC tissue samples were fixed with formalin and embedded in paraffin. A slide was coated with an anti-FAT4 mouse polyclonal antibody (1:300 dilution; Santa Cruz, USA), incubated overnight at 4 °C, washed three times with PBS and submerged in the corresponding biotinylated secondary antibody (DAKO, Shanghai, China) for 30 min. Furthermore, 3,3′-diaminobenzidine (DAB) (DAKO, Shanghai, China) was administered to develop the peroxidase reaction, and the tissue sections were also stained with hematoxylin. The IHC intensity was scored as follows: 0, 1, 2, and 3 points indicated no staining, minimal staining, moderate staining and strong staining, respectively. The percentage of positive cells was determined using a previously reported method, and the cells were divided into four groups: no positive cells, < 10% positive cells, 10–50% positive cells and > 50% positive cells [4].

### Cell proliferation, invasion and migration assays

In brief, the cells were seeded in 96-well plates (1 × 10^3^ cells/well) and cultured for 48 h. An MTT dye solution (5 mg/ml, Sigma, NY, USA) was added to each well, and the cells were incubated for another 4 h. Dimethyl sulfoxide (150 μl) was subsequently added, and the plates were placed in an ultraviolet spectrophotometer to measure the OD value of each well at 490 nm. The level of cell proliferation was examined at 1, 2, 3, 4, 5, 6 and 7 days after seeding. Cell invasion and migration were assessed using Boyden chambers (Millicell, Millipore, USA). Initially, 2 × 10^5^ cells in serum-free medium were cultured in the upper chamber of a 24-well plate, and the corresponding medium supplemented with 10% FBS was placed in the lower chamber. After 36 h of incubation, the migrated cells were fixed with 4% paraformaldehyde for 10 min, stained with 0.1% crystal violet for 10 min, air dried and counted under a light microscope. The degree of cell invasion was similarly tested using inserts precoated with Matrigel (BD Biosciences, USA) to simulate circumstances requiring invasion. Each experiment was repeated three times.

### Western blotting

Total protein from the CRC cell lines and tissues was isolated, and the protein concentrations were measured using the BCA protein assay kit (Pierce, IL, USA). The protein extracts were washed in lysis buffer, separated by 10% SDS-PAGE and transferred to polyvinylidene fluoride membranes (Millipore, Bedford, Massachusetts, USA). The membranes were blocked for 1 h in TBS-Tween (0.1%) with 2% BSA for the detection of phosphorylated proteins or in 5% skimmed milk for the detection of other proteins and then incubated with the following primary antibodies: FAT4 (1:300 dilution; Santa Cruz, USA), E-cadherin (1:1000 dilution; Santa Cruz, USA), N-cadherin (1:3000 dilution; Santa Cruz, USA), vimentin (1:1000 dilution; Santa Cruz, USA), Twist 1 (1:300 dilution; Santa Cruz, USA), β-catenin (1:5000 dilution; Santa Cruz, USA), LC3 (1:300 dilution; Santa Cruz, USA), P62 (1:300 dilution; Santa Cruz, USA), PI3K (1:300 dilution; Santa Cruz, USA), AKT (1:300 dilution; Santa Cruz, USA), p-AKT (1:300 dilution; Santa Cruz, USA), mTOR (1:300 dilution; Santa Cruz, USA), GSK-3β (1:300 dilution; Santa Cruz, USA), p-GSK-3β (1:300 dilution; Santa Cruz, USA), C-Myc (1:300 dilution; Santa Cruz, USA), unc-51-like kinase 1 (ULK1; 1:1000 dilution; Sigma-Aldrich, USA), p70(S6K) (1:1000 dilution; R&D Systems, USA), phospho-p70(S6K) (1:1000 dilution; R&D Systems, USA) and β-actin (1:1000 dilution; Beyotime, China). The primary antibodies were detected using specific secondary antibodies. The protein bands were visualized using an ECL detection reagent (Proteintech, Hubei, China), and the band densities were measured using imaging software.

### Gene knockdown or overexpression by lentiviral transduction

Lentiviral constructs for shRNA-mediated FAT4 (LV-FAT4-shRNA-puromycin) silencing and FAT4 (LV-FAT4-puromycin) overexpression were acquired from Shanghai Genechem Co, Ltd., China. The FAT4 shRNA vector sequence was as follows: (forward) 5’-GCTTTGTATAAAGTGGAGATT-3′. The three above-mentioned cell lines were cultivated in six-well plates at a density of 0.6 × 10^5^ cells per well (20–30% density) on the day prior to lentiviral transduction. LV-FAT4-shRNA-puromycin was used for the transduction of SW480 cells at a multiplicity of infection (MOI) of 40 using polybrene (10 μg/ml), which enhanced the infection efficiency (Genechem, China). Additionally, LOVO and HCT116 cells were transfected with LV-FAT4-puromycin at a MOI of 20 (LOVO) and 10 (HCT116). Moreover, two nontarget negative control viruses, puromycin-LV (Genechem, China), were used to transduce cells using the above-described methods to neutralize the influence of the viral vector. After 12 h of incubation, the medium was removed, and the appropriate fresh medium was added. After 48 h of incubation, the appropriate concentrations of puromycin were added to the cell cultures to kill cells that had not been transduced with the virus. At the indicated time points, the cells were used for protein and mRNA analyses and other assays.

### Cell immunofluorescence and confocal microscopy

The cells were cultivated on appropriate six-well coverslips, immobilized with 4% paraformaldehyde for 20 min, permeabilized with 0.2% Triton X-100 (Beyotime, Zhenjiang, Jiangsu, China) and then blocked in 10% goat serum at 37 °C for 30 min. Primary antibodies targeting LC3 (1:300 dilution; Santa Cruz, USA) and E-catenin (1:300 dilution; Santa Cruz, USA) were added to the coverslips. After incubation at 4 °C overnight, the coverslips were washed three times in PBS and incubated with the appropriate secondary antibody (SA00009-2Cy3-goat anti-rabbit IgG for LC3 and E-catenin, Proteintech) for 2 h. The nuclei were then labeled with DAPI for 10 min, and images were captured using a confocal microscope (× 600).

### Transmission electron microscopy (TEM)

For the TEM studies, the cells were exposed to RF-EMFs (1, 2, or 4 W/kg) for 24 h, harvested, washed twice with PBS (pH 7.4) at room temperature, and fixed with 2.5% glutaraldehyde. The cells were then washed three times with PBS, fixed with 1% osmic acid for 2–3 h, washed three times with PBS, dehydrated, embedded in paraffin, cut into 70-nm-thick sections using an Ultrathin slicing machine (Leica EM UC6; Leica Microsystems, Wetzlar and Mannheim, Germany), and stained with uranyl acetate-lead citrate. The cells were then observed using a transmission electron microscope (JEM1230, JEOL Ltd., Tokyo, Japan) for the detection of autophagic vacuoles.

### In vivo tumorigenesis

In our study, eight-week-old male BALB/c nude mice were purchased from the Laboratory Animal Center of Wuhan. All the animal experiments adhered to the National Institutes of Health Guide for the Care and Use of Laboratory Animals and were performed using protocols approved by the Medical Research Ethics Committee of the Second Affiliated Hospital of Nanchang University. SW480 cells (2 × 10^6^) were transduced with a FAT4 shRNA vector (LV-FAT4-shRNA-puromycin), and HCT116 and LOVO cells were transduced with a FAT4-overexpression vector (LV-FAT4-puromycin) and suspended in 100 μL of basal medium. Corresponding negative control vectors were also transduced into all three cell lines. The cells were then subcutaneously injected into the right flank of the nude mice to induce the formation of xenograft tumors. The mice were allowed to recover for 8 weeks with normal feeding and watering, and the tumors were then excised for further analysis. Specifically, the tumor volume was calculated using the formula V (mm^3^) = length×width^2^/2, and the mice were then anesthetized, euthanized, and humanely disposed.

### Statistical analyses

The data were analyzed using Prism 5 (GraphPad Software, San Diego, California, USA), and the values are presented as the means ± SEMs or SDs. A two-tailed unpaired t-test was used to compare two groups. SPSS 17.0 software (SPSS Inc., Cary, North Carolina, USA) was also used for the data analyses. A *P* value < 0.05 was assumed to indicate a statistically significant difference, and differences between *P* < 0.05 and *P* < 0.01 are denoted by single (*) and double (**) asterisks, respectively.

## Results

### FAT4 expression in CRC tissues is weaker than that in nonmalignant tissues and can inhibit cell proliferation in vitro

**Fig. 1:**
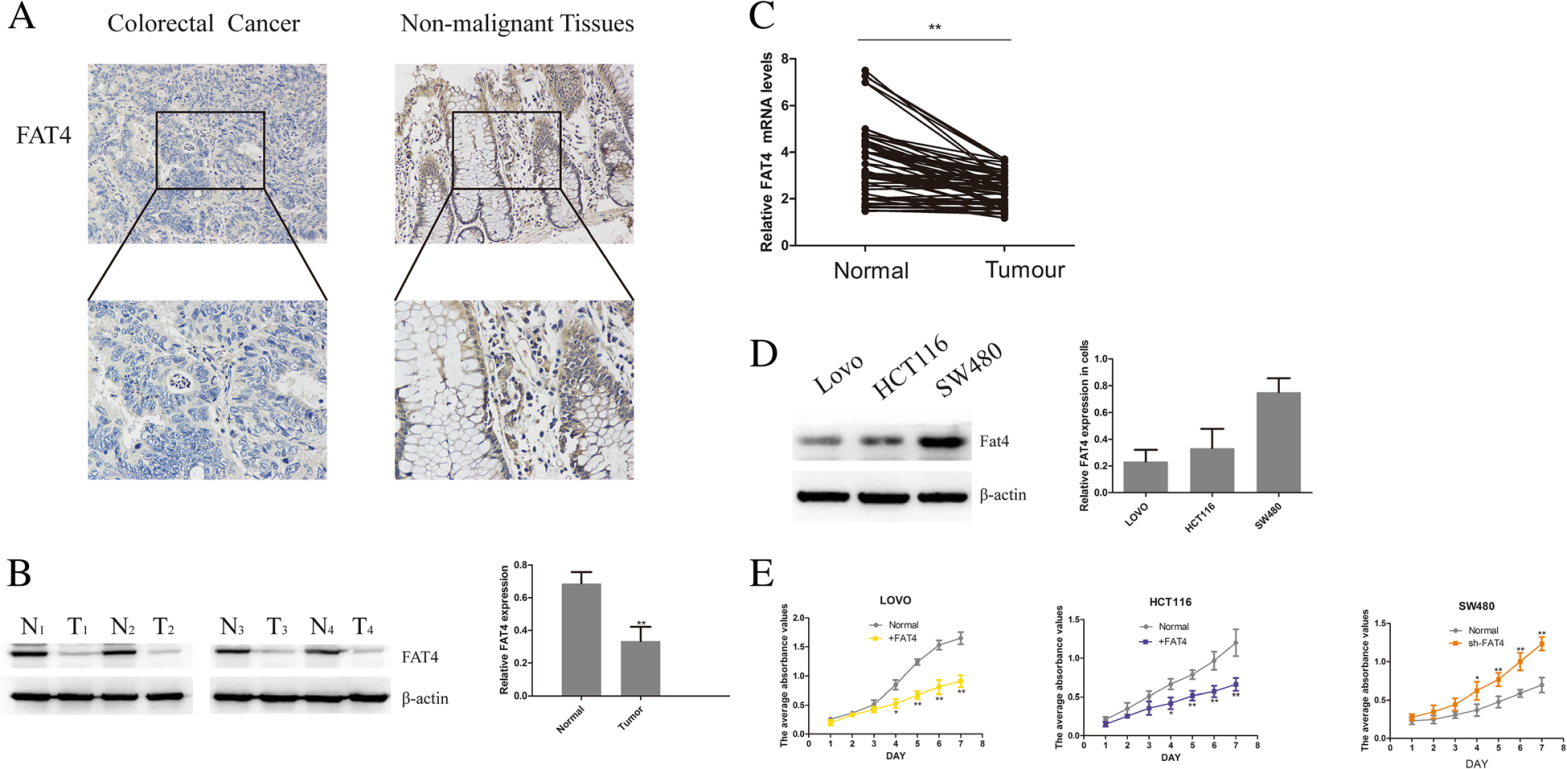
FAT4 expression in CRC tissues is weak and can inhibit cell proliferation in vitro. **a** Immunohistochemical staining of FAT4 in CRC and normal tissues. FAT4 expression was weaker in CRC tissues than in nonmalignant tissues. **b** FAT4 protein expression in representative samples of CRC tissues and paired adjacent noncancerous tissues. In CRC tissues, the expression level of FAT4 was downregulated. **c** The FAT4 mRNA expression levels in 100 CRC tissues and paired noncancerous tissues were assessed by qRT-PCR, which showed that FAT4 mRNA expression was significantly decreased in CRC tissues, as demonstrated using the 2^-ΔΔct^ method. *P < 0.05, as determined by the Mann-Whitney U-test. **d** Levels of FAT4 protein expression in three different CRC cell lines. HCT116 and LOVO cells showed low FAT4 expression, and strong FAT4 expression was detected in SW480 cells. **e** The MTT assay was used to assess the proliferation of the three CRC cell lines. During the 7-day experimental period, the HCT116 and LOVO cells in which FAT4 was overexpressed showed decreased proliferation compared with the control cells, whereas the SW480 cells transfected with shRNA FAT4 showed increased cell proliferation. *P < 0.05, as determined by Student’s t-test

In a preliminary study, we estimated the FAT4 expression levels in 100 random CRC patients, and our immunohistochemistry results revealed that FAT4 expression was decreased in the nuclei of the CRC sections compared with the nuclei in the nonmalignant tissues (Fig. 1a). In addition, a qRT-PCR analysis showed that FAT4 expression was lower in CRC tissues compared with nonmalignant tissues (Fig. 1c), and a western blotting assay confirmed that FAT4 protein expression was low in CRC tissues (Fig. 1b). To further understand the underlying relationship between low FAT4 expression and CRC, we detected the expression of FAT4 in three CRC cell lines, HCT116, SW480 and LOVO. The results revealed that SW480 cells exhibited high expression of FAT4, whereas the other two cell lines showed relatively low expression (Fig. 1d). To elucidate the pathological effects of FAT4 in CRC cells, we examined the proliferation of these cells through MTT assays and found that the proliferation capacity of SW480 cells with suppressed FAT4 expression was higher than that of control SW480 cells; however, the overexpression of FAT4 in HCT116 and LOVO cells led to a decline in the proliferation ability of these cells (Fig. 1e).

### FAT4 plays a crucial role in the EMT and autophagy in CRC

**Fig. 2:**
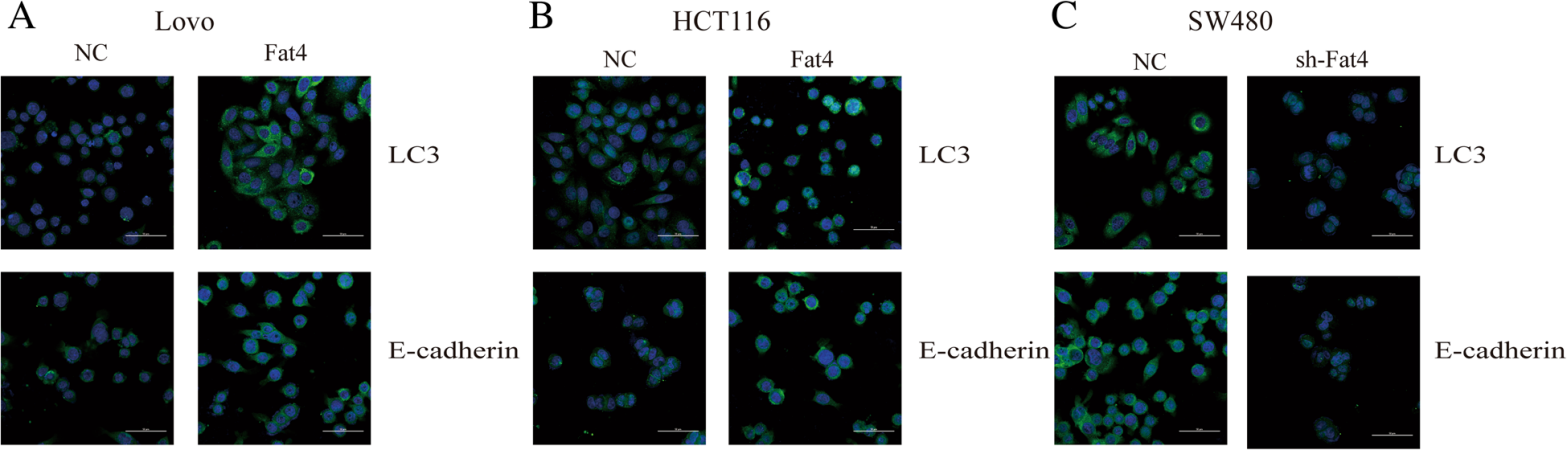
High expression levels of FAT4 could inhibit the EMT and induce autophagy in CRC. **a**-**c** Immunofluorescence staining was performed to detect the expression of FAT4 in cells transduced with FAT4 shRNA or a FAT4-overexpression vector compared with that in cells transfected with the negative control. The results revealed that LC3 expression was elevated in the FAT4-overexpressing LOVO and HCT116 cells, whereas FAT4 knockdown inhibited the expression of LC3 in SW480 cells. **a**-**c** In addition, in HCT116 and LOVO cells, FAT4 overexpression increased E-cadherin expression, thereby inhibiting the EMT, whereas in SW480 cells, FAT4 knockdown suppressed E-cadherin expression

**Fig. 3:**
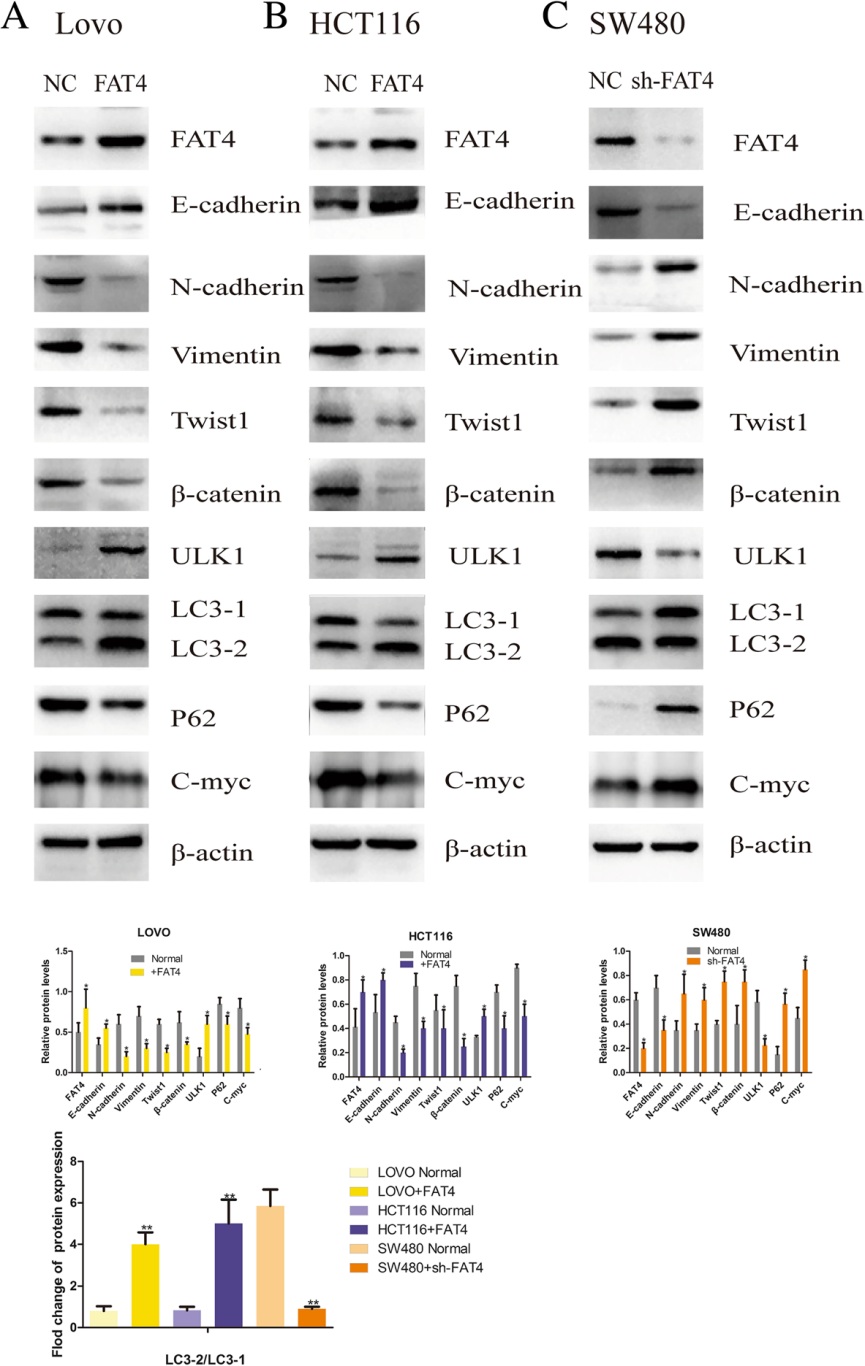
FAT4 can regulate the EMT and autophagy in CRC. FAT4 modulates the expression of EMT and autophagy markers. **a**-**c** We performed a western blotting assessment of FAT4, β-catenin, E-cadherin, N-cadherin, vimentin, C-Myc, Twist1, ULK1, LC3 and P62 protein expression in three cell lines with modified FAT4 expression. In FAT4-overexpressing HCT116 and LOVO cells, the expression levels of N-cadherin, vimentin, P62, C-Myc, β-catenin and Twist1 were decreased, whereas E-cadherin and ULK1 expression was enhanced. The LC3-II/LC3-I ratio was higher in the FAT4-overexpressing HCT116 and LOVO cells. SW480 cells transfected with shRNA FAT4 exhibited the opposite outcomes. *P < 0.05, as determined by Student’s t-test

**Fig. 4:**
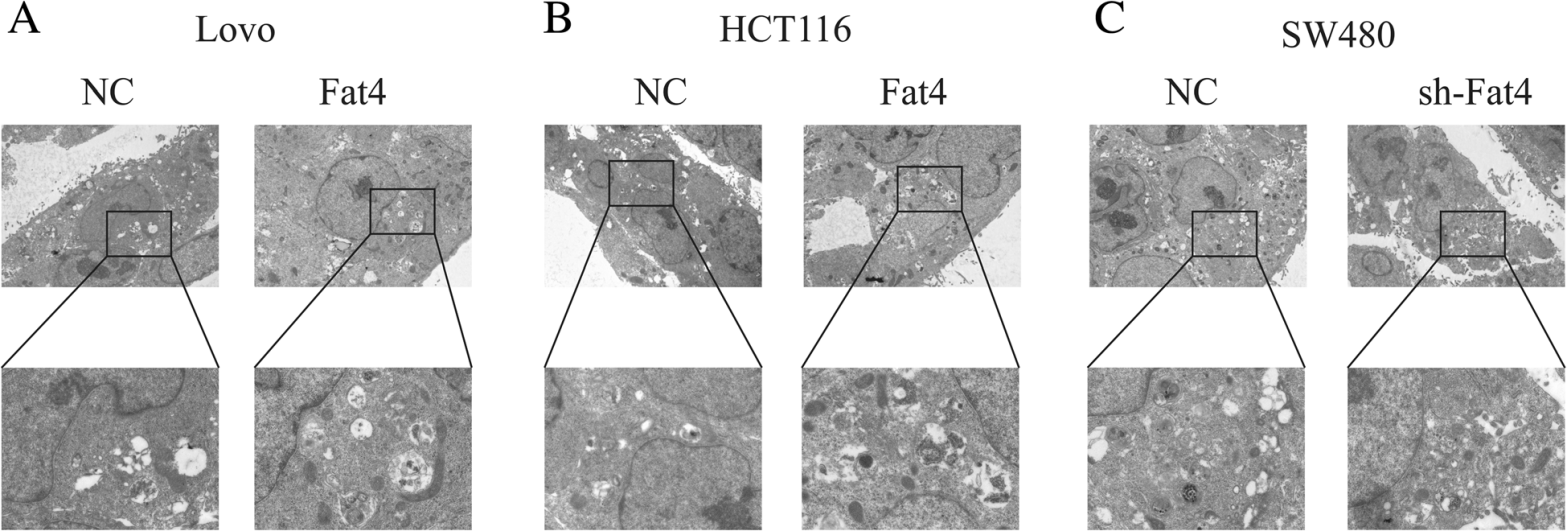
Effects of FAT4 on autophagosomes in CRC cells. **a**, **b** Autophagosomes were observed by TEM in CRC cells. The results showed increased autophagosomes in FAT4-overexpressing HCT116 and LOVO cells compared with the control cells. **c** The inhibition of FAT4 expression in SW480 cells decreased the number of autophagosomes. Magnification, × 1500 and 5000

In line with the results of our previous experiment, HCT116 and LOVO cell lines, which manifested relatively low nascent FAT4 expression, were transduced with FAT4-overexpressing constructs, and SW480 cells were transduced with shRNA to knockdown FAT4 expression. It is well accepted that E-cadherin, N-cadherin and vimentin are considered classical EMT markers, and the immunofluorescence and western blotting results demonstrated that E-cadherin expression was enhanced in FAT4-overexpressing HCT116 and LOVO cell lines and dysregulated in FAT4-knockdown SW480 cells compared with the levels in the corresponding untreated cells. In contrast, N-cadherin, vimentin, C-Myc, β-catenin and Twist1 expression showed an opposite trend (Fig. 2a-c, Fig. 3a-c). Furthermore, the function of FAT4 in gastric cancer was previously studied [3]. The EMT requires cancer cells to survive independently from the primary tumor site without a nutrient support system, and as a result, these cells might be more sensitive to autophagy [5]. Autophagy might play a crucial role in cell invasion and migration. To investigate this phenomenon, we further explored the function of FAT4 in autophagy. The overexpression of FAT4 significantly elevated the LC3-II/LC3-I ratio and ULK1 level and decreased P62 expression, as indicated by western blotting. In contrast, the knockdown of FAT4 in SW480 cells decreased the LC3-II/LC3-I ratio and ULK1 level and promoted the expression of P62 (Fig. 3a-c). Through immunofluorescence, we observed that FAT4-overexpressing HCT116 and LOVO cells showed strong LC3 expression, whereas FAT4-knockdown SW480 cells exhibited weak LC3 expression. E-cadherin showed the opposite results in the transduced cells: high expression in the FAT4-overexpresing HCT116 and LOVO cells and low expression in the FAT4-knockdown SW480 cells (Fig. 2a-c). Additionally, as observed by TEM, autophagosome formation was significantly induced in the HCT116 and LOVO cells transduced with the FAT4-overexpressing constructs, whereas the inhibition of FAT4 expression in SW480 cells resulted in a decreased formation of autophagosomes (Fig. 4a-c). Together, these findings provide strong evidence showing that FAT4 plays a crucial role in the EMT and autophagy in CRC.

### The regulatory effects of FAT4 on autophagy and the EMT are partially due to the PI3K-AKT signaling pathway

**Fig. 5:**
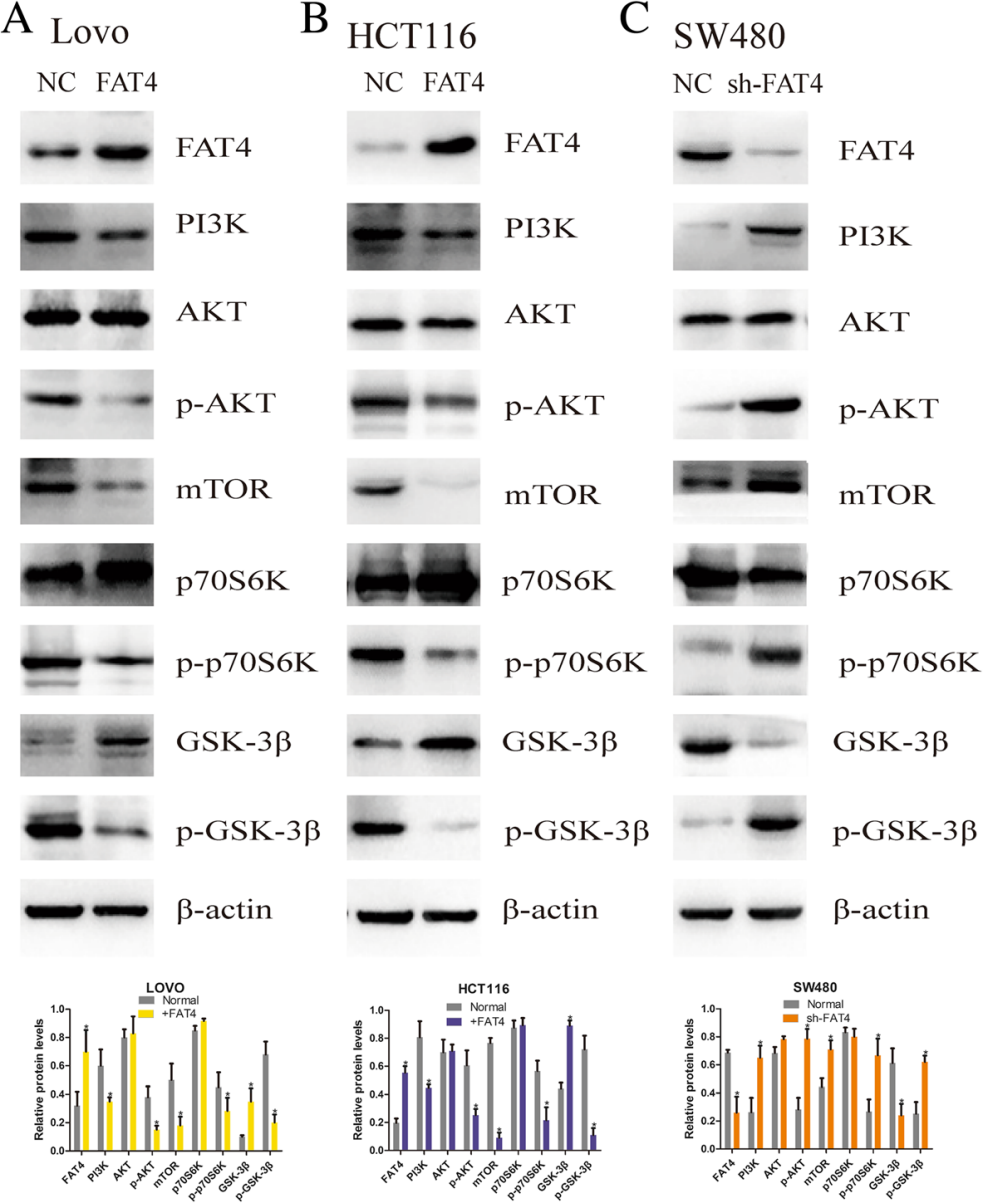
FAT4 induces autophagy and inhibits the EMT through the PI3K-AKT signaling pathway. **a**-**c** The levels of relevant signaling pathway proteins in cells transduced with FAT4 shRNA or a FAT4-overexpression vector were examined by western blotting, and the images were compared with those of NC cells. As shown, FAT4 overexpression visibly decreased the expression levels of PI3K, p-AKT, mTOR, p-p70S6K and p-GSK-3β and induced GSK-3β. In SW480 cells, the knockdown of FAT4 increased the expression of PI3K, p-AKT, mTOR, p-p70S6K and p-GSK-3β and reduced GSK-3β expression compared with the levels observed in the control cells. β-Actin was used as the internal control. *P < 0.05, as determined by Student’s t-test

**Fig. 6:**
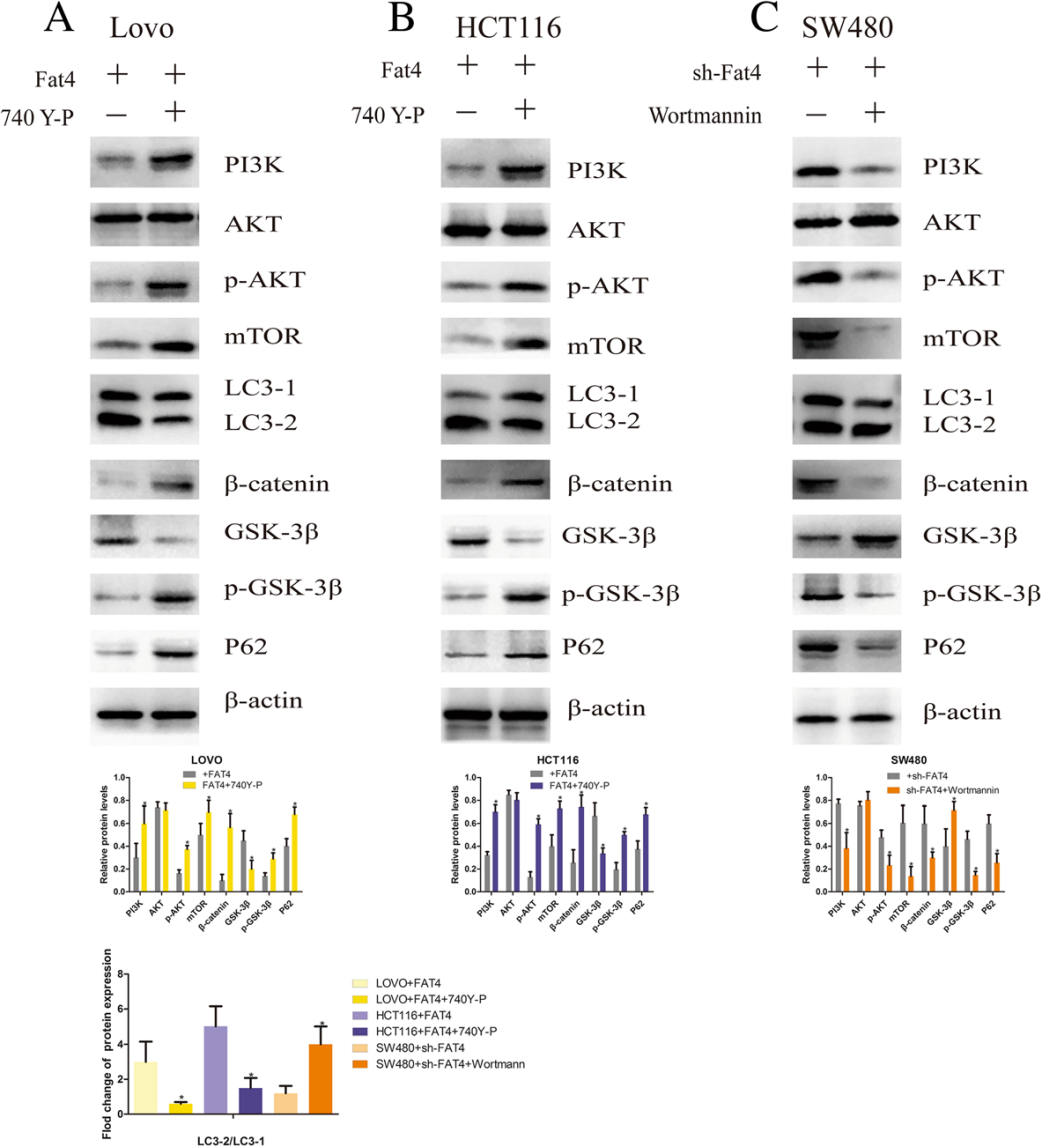
FAT4 regulates autophagy and the EMT by regulating the level of PI3K. **a**-**c** The levels of various factors in cells transduced with a target gene or negative control and treated with either the PI3K inhibitor wortmannin or the PI3K activator 740Y-P were examined by western blotting. The treatment of FAT4-overexpressing cells with 740Y-P increased the expression of PI3K, p-AKT, mTOR, β-catenin and p-GSK-3β but decreased the expression of GSK-3β. In these cells, the LC3-II/LC3-I ratio, which is used as an autophagy marker, was reduced, and P62 expression was increased; these changes result in the inhibition of autophagy. The treatment of sh-FAT4 SW480 cells with the PI3K inhibitor wortmannin reduced the expression of PI3K, p-AKT, mTOR, β-catenin and p-GSK-3β, increased the expression of GSK-3β and LC3-II/LC3-I ratio, and decreased the expression of P62. *P < 0.05, as determined by Student’s t-test

Based on the above-described results, we hypothesized that FAT4 played a significant role in the EMT and autophagy to affect the tumor characteristics of CRC cells and that this activity partially relies on the PI3K-AKT signaling pathway. Based on these assumptions, we aimed to detect the relevant effectors within this pathway through western blotting. First, we evaluated PI3K expression in these three cell lines, and the results indicated that PI3K expression was notably downregulated in the two FAT4-overexpressing cell lines compared with the FAT4-knockdown SW480 cells (Fig. 5a-c). Furthermore, the level of p-AKT expression was reduced after FAT4 overexpression, and SW480 cells with suppressed FAT4 expression showed increased levels of this protein (Fig. 5a-c). In addition, as demonstrated by the western blotting assays, the FAT4-overexpressing HCT116 and LOVO cells exhibited higher levels of GSK-3β and lower levels of mTOR expression compared with those in the corresponding control cells. The level of p-p70S6K expression was reduced following FAT4 overexpression, and the suppression of FAT4 expression in SW480 cells increased the expression of p-p70S6K (Fig. 5a-c). However, the knockdown of FAT4 led to decreased GSK-3β and increased mTOR expression, and the phosphorylation of GSK-3β (p-GSK-3β) showed the opposite trend to GSK-3β, which revealed that the regulatory effects of FAT4 can be partially attributed to the PI3K-AKT-mTOR and PI3K-AKT-GSK-3β signaling pathways (Fig. 5a-c). Although the carcinostatic function of FAT4 is specific, further investigation is urgently needed to illustrate the mechanism through which FAT4 affects the EMT and autophagy in tumors via the PI3K-AKT signaling pathway. Thus, we applied wortmannin, a PI3K-AKT signaling pathway inhibitor, and the activator 740Y-P to the cells showing stable FAT4 knockdown or overexpression and evaluated the expression of PI3K, AKT, p-AKT, mTOR, GSK-3β, p-GSK-3β and β-catenin by western blot analysis. The results showed that in the FAT4-overexpressing HCT116 and LOVO cells, the expression levels of PI3K, p-AKT, mTOR, p-GSK-3β and β-catenin were increased after treatment with the PI3K activator 740Y-P, but these levels were reduced in the FAT4-knockdown SW480 cells after treatment with the PI3K inhibitor wortmannin (Fig. 6a-c). In contrast, GSK-3β showed the opposite results (Fig. 6a-c). In addition, the LC3-II/LC3-I expression ratio was decreased in the FAT4-overexpressing HCT116 and LOVO cells after 740Y-P treatment, but the levels of P62 were augmented in these cells (Fig. 6a-c). Additionally, wortmannin treatment inhibited the PI3K-AKT signaling pathway, leading to reduced expression of PI3K, p-AKT, mTOR, β-catenin, and p-GSK-3β, whereas the GSK-3β level was increased in FAT4-silenced SW480 cells. In contrast, the LC3-II/LC3-I expression ratio was increased after wortmannin treatment, resulting in inhibition of the PI3K-AKT-mTOR signaling pathway and thereby the induction of autophagy, as demonstrated by a decreased level of the negative autophagy marker P62. Considering these results, we conclude that the regulatory effects of FAT4 on autophagy and the EMT can be partially attributed to the PI3K-AKT-mTOR and PI3K-AKT-GSK-3β signaling pathways.

### Autophagy induction regulates the migration and invasion of CRC cells

**Fig. 7:**
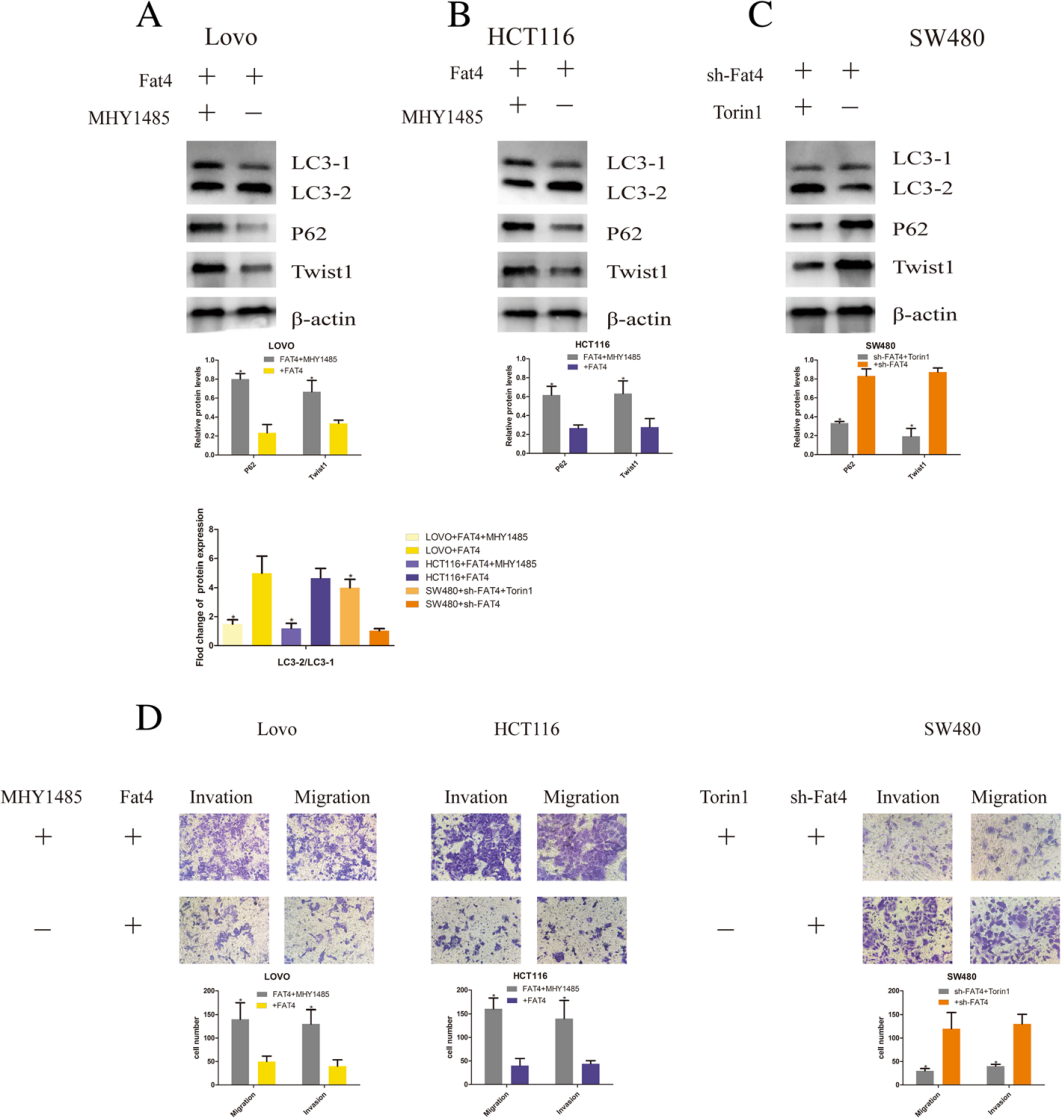
Autophagy induction regulates the migration and invasion of CRC cells. **a**-**c** In FAT4-overexpressing HCT116 and LOVO cells, the expression levels of P62 and Twist1 were increased after treatment with the autophagy inhibitor MHY1485, but these levels were reduced in FAT4-knockdown SW480 cells treated with the autophagy activator Torin 1. In addition, the LC3-II/LC3-I expression ratio was decreased in FAT4-overexpressing HCT116 and LOVO cells treated with MHY1485 and increased in FAT4-knockdown SW480 cells treated with Torin 1. **d** Transwell assays revealed that cell migration and invasion were promoted after autophagy was blocked in FAT4-overexpressing HCT116 and LOVO cells by treatment with the autophagy inhibitor MHY1485. In FAT4-knockdown SW480 cells, the promotion of autophagy by treatment with the autophagy activator Torin 1 inhibited cell migration and invasion. *P < 0.05, as determined by Student’s t-test

To confirm the inhibitory function of autophagy in cell chemotaxis, we analyzed the migration and invasion capability of CRC cells transduced with FAT4-overexpressing or FAT4-knockdown constructs. Our previous results demonstrated that FAT4 can promote autophagy and inhibit the migration and invasion of CRC cells via the PI3K-AKT-mTOR and PI3K-AKT-GSK-3β signaling pathways. Additionally, we questioned whether FAT4 can reduce the EMT by increasing the activity of autophagy. The study conducted by Gugnoni indicated that accumulated P62 might ubiquitinate Twist1 to promote the stabilization of Twist1 and the EMT in papillary thyroid carcinomas [4]. However, in CRC, whether FAT4 can regulate autophagy to influence the EMT needs to be confirmed. Thus, we applied MHY1485, an autophagy inhibitor (mTOR promoter), and the activator Torin 1 (mTOR inhibitor) to the cells showing stable FAT4 overexpression or knockdown and evaluated the expression of LC3, P62 and Twist1. The results showed that in the FAT4-overexpressing HCT116 and LOVO cells, the expression levels of P62 and Twist1 were increased after treatment with the autophagy inhibitor MHY1485, but these levels were reduced in FAT4-knockdown SW480 cells treated with the autophagy activator Torin 1. In addition, the LC3-II/LC3-I expression ratio was decreased in the FAT4-overexpressing HCT116 and LOVO cells following MHY1485 treatment, and this ratio was increased in the FAT4-knockdown SW480 cells treated with Torin 1. The inhibition of autophagy is associated with an increased level of P62, which prevents the degradation of Twist1. Transwell assays revealed that cell migration and invasion were promoted after the attenuation of autophagy in the FAT4-overexpressing HCT116 and LOVO cells treated with the autophagy inhibitor MHY1485, whereas the FAT4-knockdown SW480 treated with the autophagy activator Torin 1 showed poor cell migration and invasion (Fig. 7a-c). These findings demonstrate that autophagy can partially inhibit the migration and invasion of CRC cells, and this effect can be partly attributed to the PI3K-AKT-mTOR signaling pathway.

### FAT4 modulates CRC tumorigenesis in vivo

**Fig. 8:**
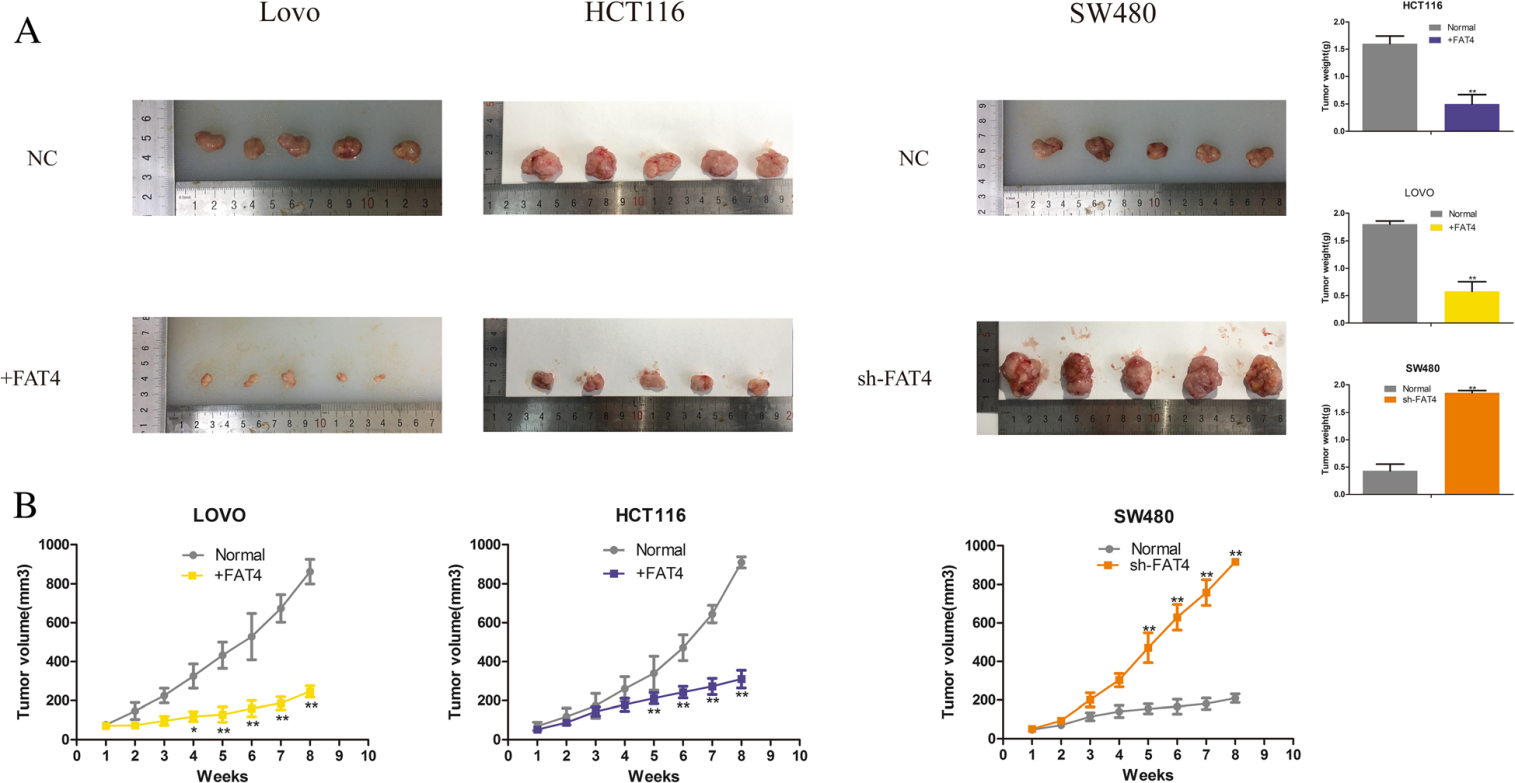
Effect of FAT4 knockdown and overexpression on tumorigenesis in vivo. **a** Photographs of tumors excised from mice injected with normal cells or cells with modified FAT4 expression (*n* = 5 per group). After 56 days, the mice were sacrificed, and the tumors were weighed. **b** Tumor growth curves for mice injected with normal cells or cells with modified FAT4 expression. *P < 0.05, as determined by Student’s t-test

After gaining abundant acknowledgement of the in vitro effects of FAT4, we sought to further elucidate the tumor suppressor role of FAT4 during tumor growth in vivo. Thus, we constructed a tumor xenograft model. Specifically, tumor xenografts were successfully obtained with FAT4-overexpressing HCT116 and LOVO cells, and the tumor volumes obtained with these cells were smaller than those obtained with the control cell lines. In contrast, the tumors derived from the FAT4-silenced SW480 cells were increased compared with those obtained with the non-transduced SW480 cells. The tumor growth curve is demonstrated in the right panel of Fig. 8b. Furthermore, throughout the growth process, the average tumor weight was typically obtained in the groups with reduced FAT4 expression, whereas the FAT4-overexpressing HCT116 and LOVO cells yielded tumors with relatively lighter weights throughout the growth process (Fig. 8a). In conclusion, these data are consistent with the results from the in vitro cell proliferation assay and indicate that FAT4 is associated with CRC progression and tumorigenesis in vivo.

## Discussion

CRC is a common human malignancy, and an in-depth understanding of its molecular mechanisms is urgently needed [1]. In this study, we aimed to carefully determine the role of the FAT4 gene in CRC development and to identify the associated signaling mechanisms. The EMT is a physiological process that increases the invasion and migration abilities of cells and has been found to be important for tumor metastasis and development in numerous cancers [6]. The expression levels of some molecular markers could reveal the extent of the EMT because reduced E-cadherin expression and upregulated N-cadherin and vimentin expression significantly induce the EMT [20, 21]. Previous studies have shown that FAT4 can enhance the expression of E-cadherin and inhibit the expression of N-cadherin and vimentin to inhibit the EMT. Twist1, a significant mediation factor downstream of β-catenin, is involved in promoting the EMT [4]. Additionally, Twist1 induces a decrease in E-cadherin-mediated cell-cell adhesion to promote the EMT [22]. After β-catenin accumulates in the cytoplasm, it translocates to the nucleus and forms an active complex with LEF (lymphoid enhancer factor) and TCF proteins to induce the transcription of downstream target genes [6]. In addition, FAT4 might decrease the levels of β-catenin and then downregulate Twist1 expression to suppress CRC development, as demonstrated in the study of gastric cancer conducted by Cai [4]. The EMT enables cancer cells to survive independently from the primary tumor site without a nutrient support system, and thus, these cells might be show some increased sensitivity to autophagy [7].

Autophagy is a lysosomal degradation pathway that engulfs, digests and recycles intracellular proteins and organelles to produce energy [23], and this process could also limit cell damage and sustain viability under detrimental conditions. Compared with normal cells, cancer cells face more environmental and intrinsic metabolic stresses and might be notably more dependent on autophagy [24]. To balance cellular degradation and the maintenance of functional integrity, autophagy is selective and leads to mitophagy [7]. The increase in FAT4 expression observed in CRC cells could enhance the levels of LC3 and ULK1 and decreasing P62 accumulation, as demonstrated by our western blotting results, which indicates that FAT4 might promote autophagy in CRC. After its processing, LC3 plays a significant role in the formation of autophagosomes through a mechanism related to the autophagosome membrane. This protein is found in two forms, LC3-I and LC3-II: LC3-I is cytosolic, whereas LC3-II is present both inside and outside autophagosomes [25, 26]. In addition, LC3-II might regulate the formation of autophagosomes and control the number of autophagosomes [27, 28]. In addition to autophagy-promoting serine-threonine kinases, the level of ULK1 is critical for the regulation of autophagy [29, 30], and autophagy can also be regulated by other nutrient-sensitive kinases, such as TORC1 [31, 32]. Self-phosphorylated ULK1 can phosphorylate both FIP200 and Atg13, which might promote translocation of the entire complex to induce autophagy [33–35]. Moreover, P62 and ubiquitin-containing protein aggregates can be induced to form, and these can combine with Atg8 in autophagic membranes to instigate the breakdown of autophagosomes [36, 37]. P62 accumulation directly induces elevation sin oxidative stress and mitochondrial damage, and an increase in P62 expression results in autophagy deficiency [7].

The EMT and autophagy are linked through a disputed and still unclear relationship in CRC. Some studies have indicated that autophagy plays a significant role in promoting the invasion and migration of hepatocellular carcinoma cells by inducing the EMT [14, 15]. Autophagy might protect against tumor initiation by decreasing genomic instability and helping remove damaged organelles and proteins [38]. However, a recent study showed that upregulation of the autophagic machinery might limit the EMT phenotype by increasing the instability of key EMT proteins [16]. In this study, we compared the molecular and cellular functions of FAT4 between autophagy and the EMT and observed that Twist1 is downregulated in CRC cells treated with the mTOR inhibitor Troin1 and that Twist1 expression is upregulated by treatment with the mTOR promoter MHY1485, which can decrease the level of E-cadherin. The results related to the E-cadherin levels showed a consistent trend, which indicated that autophagy might inhibit the EMT in CRC cells. Under autophagy-deficient conditions, cell proliferation and migration are increased through the accumulation of P62 and the subsequent ubiquitination of Twist1 to promote its stabilization [39]. Twist1 can be removed through both autophagy and proteasomal degradation. However, in autophagy-deficient cells, the degradation of Twist1 by the autophagosome is inhibited [40], supporting an important role for autophagy in EMT repression.

**Fig. 9:**
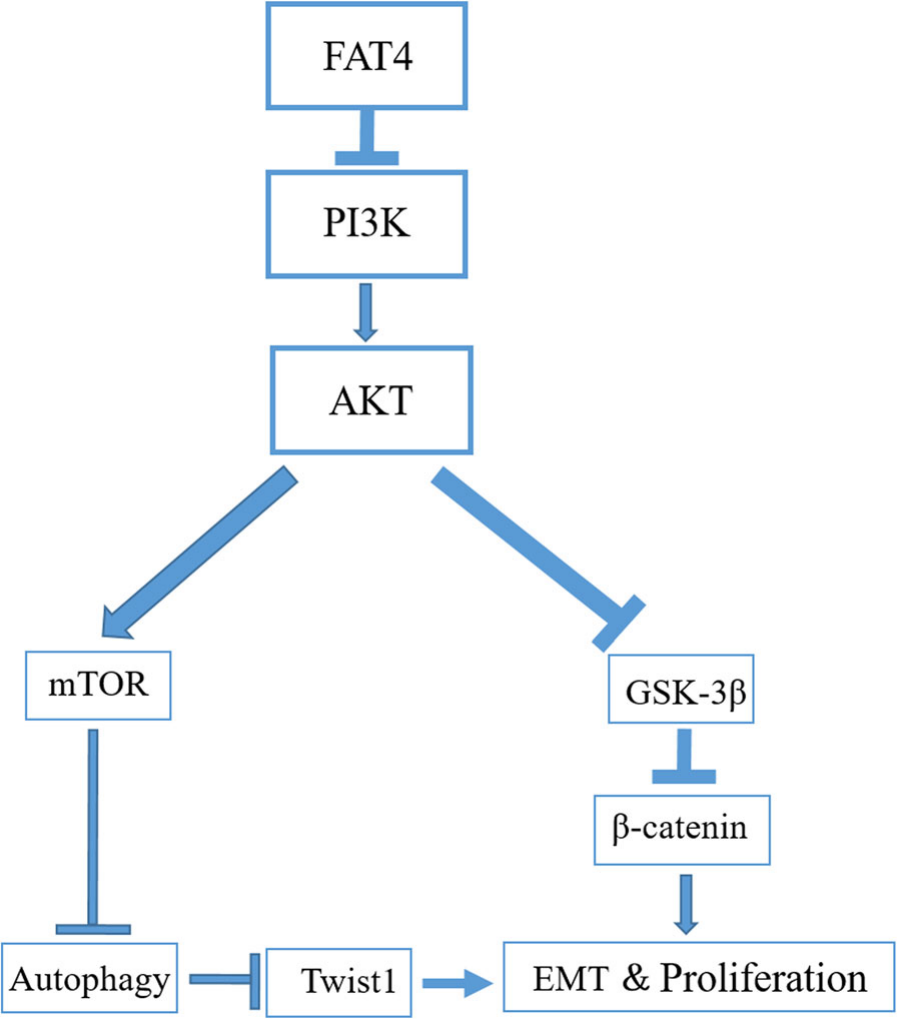
Schematic diagram of the mechanism through which FAT4 inhibits the EMT and promotes autophagy in CRC via the PI3K/AKT/GSK-3β and PI3K/AKT/mTOR signaling pathways. Through the overexpression and silencing of FAT4 and PI3K, we demonstrated that FAT4 might negatively regulate PI3K to inhibit cancer development. In addition, FAT4 can inhibit the EMT by promoting autophagy and decreasing the level of Twist1

Autophagy can be controlled by the downstream targets of mTOR, a significant signaling node of the PI3K/AKT/mTOR pathway [41, 42]. PI3K phosphorylates PIP2 to produce PIP3, and PIP3 can induce the phosphorylation of the serine residues 308 and 473 (Ser308 and Ser473, respectively) of AKT. p-AKT (phosphorylated AKT) can activate mTOR by inhibiting TSC2 and TSC. In addition, mTOR might promote mRNA translation by phosphorylating S6K1 and then p70S6K to promote the adhesion of ribosomes to the endoplasmic reticulum, thereby preventing the formation of autophagic membranes and inhibiting autophagy activity [43, 44]. The corresponding results from our study show that FAT4 might inhibit the levels of PI3K and decrease the expression of p-AKT and mTOR to promote autophagy. Additionally, the PI3K/AKT pathway, particularly its substrates, plays a crucial role in the EMT [45, 46]. p-Akt can phosphorylate GSK-3β at Ser9 and inhibit its kinase activity to decrease GSK-3β [47, 48], a major member of the destruction complex (APC, GSK-3β and Axin-2), which is capable of downregulating both T-β-catenin and N-β-catenin in cells [49–51]. FAT4 can increase the expression levels of constituents of this complex by subsequently downregulating both T-β-catenin and N-β-catenin expression in gastric cancer [4]. Moreover, the in vivo study of the function of FAT4 yielded similar results (Fig. 9).

## Conclusions

In summary, our data reveal that FAT4 is a tumor suppressor in CRC. Moreover, FAT4 silencing inhibits CRC cell autophagy and stimulates the invasion and migration of these cells as well as the EMT, whereas the overexpression of FAT4 yields the opposite results and increases autophagy. Furthermore, the stimulatory effects of FAT4 on autophagy occur through the upregulation of LC3 and the downregulation of P62 and through the effects of FAT4 on the EMT, as evidenced by the detected changes in the expression levels of E-cadherin and Twist1. Moreover, an increase in FAT4 leads to a reduction in xenograft tumor growth in vivo, whereas the opposite outcome was obtained with FAT4 knockdown. Therefore, we conclude that FAT4 regulates the activity of PI3K to promote autophagy and inhibit the EMT, and these effects are partly achieved through the PI3K/AKT/mTOR and PI3K/AKT/GSK-3β signaling pathways. We anticipate that this study will provide a basis for establishing new strategic approaches for the development of effective CRC therapies.

## Abbreviations

ATP: Adenosine triphosphate
BCA: BCA protein assay kit
BD: Matrigel
CRC: Colorectal cancer
DAB: Diaminobenzidine
ECL: Enhanced chemiluminescence
EGFR: Epidermal growth factor receptor
EMT: Epithelial-to-mesenchymal transition
FBS: Fetal bovine serum
IHC: Immunohistochemistry
LEF: Lymphoid enhancer factor
LV: Lentiviral
MOI: Multiplicity of infection
OD: Optical density
p-AKT: Phosphorylated AKT
PBS: Phosphate buffer solution
p-GSK-3β: Phosphorylated GSK-3β
PI3K: Phosphoinositide 3-kinase
qRT-PCR: Quantitative real-time PCR
SD: Standard deviation
SDS-PAGE: Sodium dodecyl sulfate polyacrylamide gel electrophoresis
shRNA: Short hairpin RNA
TEM: Transmission electron microscopy
ULK1: Unc-51-like kinase 1
